# Criticisms: Unjust, Unliberal and Unprofessional

**Published:** 1906-11

**Authors:** H. Herbert Johnson

**Affiliations:** Macon, Ga.


### CRITICISMS: UNJUST, UNLIBERAL AND UNPROFESSIONAL.

By H. Herbert Johnson, D. D. S., Macon, Ga.

Georgia State Dental Society, 1906.

For appearing before you with nothing more than a new presentation of an old subject, which has already been so often written upon, I find an excuse which seems to somewhat justify the charge, in the fact that the gospel of Christ has been continuously preached for about nineteen hundred years, and though there are today more churches, bibles and hard worked preachers, as well as other evidences of Christianty in existence than at any other period of the world's history, strange to record, there yet remains a large number of unconverted sinners.

Man, in all matters of civilized society is usually guided by feelings of conscientiousness. In his improved and cultivated nature he loves the right and hates the evil, yet in matters generally he needs to be constantly reminded of his duty and short comings that he may walk upright continuously. "He walks theoretically and instinctively guided by his feelings, but in *practical* walking he needs the constant admonition ever before him, 'keep to the right.'"

Then, again, while the subjects of ethics, fraternalism, brotherly love, etc., have been fully, forcefully and repeatedly presented to us in the past, there are yet few among us whose daily conduct and conversation is so correct and far above criticism as to render it unnecessary to, not once, but again and again, agitate and discuss the art of right living, that we may continually have our minds refreshed and our digressions and duties brought to view.

While the errors as I shall discuss them are not a palpable violation of the *code* of *ethics,* which are the rules of law adopted by this Society, yet they are a direct violation of *ethics* when viewed from a broad and liberal standpoint. I will not, therefore, discuss the *code* of *ethics* or violations of the same, because that is a written law to which we are amenable, but in stead, I will discuss violations of ethics, from a moral, Christian and humane standpoint, in the manner in which it affects our brethren in the profession as well as that which is still more vitally important, our clientele.

It would be but natural for the question to, therefore, arise in the minds of some as to what would be a correct definition of ethics.

The accepted authorities tell us that ethics is a moral science that treats of the moral agent as an intelligent and free being possessed of conscience; "that it is the science that offers a rational explanation of the ideas of rightness and oughtness, and that it deals with the life of free personal beings under these conceptions; that it is the science of human duty, of right and of right character and conduct; that it is the principles of the foundation of the right regulation of conduct."

Dr. G. S. Dean says, "ethics is an art and not a science, that it is the art of forming, or of guiding, or of forming and guiding, human character. What, now, is human character– In the special sense in which the word is practically used, the human character is that portion of the affinities or emotions of the man which render him human, which raise him above the brutes—the social affinities—the sentiments which create or affect the human sense, no society, no character, therefore, no society, no ethics."

Ethics, then, being a science, to be sufficiently grasped by the now receptive but originally blank human mind, must be both taught and studied, having the insatiable and burning desire present for a thorough and full understanding; if ethics be an art, by long cultivation throughout generations the tendency to a high and perfect state may be at last inherited, which added to by continued study and practice may become natural and fixed in the habits of the individual.

Dr. Bogue calls ethics "that intangible but quite actuality called morals. The lower down in the scale of humanity the less the knowledge of morals, because there are no morals to have knowledge of; but as civilization advances, ethics, or the knowledge of morals becomes more widely disseminated, until we reach a distinctly Christian community, when the science crystalizes itself into the aphorism promulgated by the Divine Healer, 'as ye would that others should do onto you, do ye even so unto them.' "

From the foregoing brief but concise explanation of the definition we can easily understand the difficult problem which confronts us when we attempt to maintain the critical position of a strictly ethical practitioner; and the thought is still further impressed upon us as to the very limited number in our ranks who are really entitled to the honorable name of ethical, though its application is quite commonly and thoughtlessly applied.

It, therefore, behooves most of us who would desire to have the term ethical deservedly written in our obituaries or recorded in our biographies to bestir ourselves that we may take on sufficient moral training to acquire the art or cultivate the science. That there is a distinct need of such cultivation throughout the profession is very apparent, and probably the most dangerous and damaging practice in violation of ethics, is that kind referred to in the caption of the paper, for this deals with a part very subtle in its character and of almost daily occurrence.

How natural and how human to fall into the error of attempting to build up our own name, reputation or character by damaging or even completely tearing down that of another.

Said the poet:

"Who steals my purse steals trash; 'tis something, nothing.
'Twas mine, 'tis his and has been slave to thousands;
But he that filches from me my good name
Robs me of that which not enriches him
But makes me poor indeed."

Probably the most prolific source of erring lies with those cases in practice which we are constantly called upon to examine, and through an imperative but not a *desired* duty, are required to pass judgment upon work done by others. Many times we must be extremely guarded, even *tactful* to prevent doing an injustice, nay direct injury to a brother practitioner, whom it may be that we easily recognize as equal to ourselves in judgment and skill. *Here it is* that the unscrupulous, unethical and immoral find their most acceptable opportunity, and are delighted to take advantage of it openly and boldly. Going many times far out of the way to criticise, magnify and call special attention to defects, really, of no special consequence, and as I have been informed are often guilty of pulling out fillings or tearing off crowns that are beyond their skill to replace with the same degree of perfection. In this way openly and selfishly attempting to damage the reputation of a man whose skill is probably *many times* greater than their own, to say nothing of the injury wrought to the profession itself through such deceit and misrepresentation. Such baseness makes criminal guilt, which though, not amenable to the laws of the land, is more low and damnable than that of robbing and stealing. *Here it is,* also, that criticisms, unjust and unprofessional may be indulged in by those who *mean well,* and make some pretense to ethical conduct, through thoughtless and careless speech, a shrug of the shoulder, a superior and wise look, or even by intentional silence when a slight explanation is justly due and would easily set the mind of the patient right towards the other man.

*Here it is* that the *truly ethical* should be forever on guard, using every artful tact to shield the reputation of a brother, whose skill or the honesty of whose purpose has been questioned or assailed by an irate patient. When it becomes necessary, in passing judgment upon a piece of failing or apparently defective work, the truly ethical man will give an honest opinion as to the stablilty of the work, for this much is due the patient, but if he has the true element of sympathy and brotherly love in his heart, he will seek rather to explain away the cause of the failure, so as to satisfy the mind of the patient as to the honest and skillful attention they had received at the hands of the other dentist. But is this course always pursued? It is lamentable to state that it is not. With Burns we mourn,

> "That man, whose heav'n erected face
> The smiles of love adorn,
> Man's inhumanity to man
> Makes countless thousands mourn."

It is our bounden duty to keep strict guard over our feelings and be very sure to mediate carefully and conscientiously, lest some petty profesional jealousy—some lurking brutality—should cause some concealed criticism to find lodgment in our breast against the success of our brother. Too often it is the case, "out of the fullness of the heart the mouth speaketh." Had this rankle been kept out of our *minds* there would be no possibility of a probable slip of the tongue at an unguarded moment.

There is no possible way by which we may *know* the circumstances or disadvantages attending the rendering of the service in question, as patients themselves are always purposely silent on this point.

There may have been very good and excusable reasons, such as:

The operation was unavoidably hurried.

The patient unusually sensitive to pain at this very time from ill health, loss of sleep or other cause.

The dentist himself may have been sick, nervous, worried, tired or from some such cause rendered quite incapacitated for work at this particular hour, or day, failing to measure up to his usual ability and skill; for be it known that our work is the most taxing and nerve racking of any labor that human beings may engage in.

Want of care and cleanliness on the part of the patient may have caused serious damage to the foundation of the work.

The health of the patient may have undergone a subsequent change which very much deranged the organic functions of the body and in consequence wrought great destruction to the teeth, but may have again in due time improved so that no trace of such condition was at this time observable or indicated.

Indeed these probable causes could be multiplied *ad infinitum,* but they are so very apparent that it is a useless waste of time to recite them. One thing of importance, however, we must at all times bear in mind, and that is that it matters not how unskillfully a piece of work coming under supervision may appear from its present condition, at some time during our career, we have had the misfortune to send out from *our* hands, and *our* offices, work that was equally as bad if not worse. Therefore, remember that if we fail to observe our ethics and through thoughtlessness, malice aforethought or otherwise, criticise our brother harshly, the time will surely come when the same opportunity will come to him and the science of ethics and the milk of human kindness must be indeed deeply rooted in his soul if he declines to avail himself of this splendid opportunity.

That we can ever make ethical practitioners, by any available means, out of the entire profession is indeed too much to hope for.

The human race having ascended to civilization from a barbaric condition or state, which it has taken centuries to accomplish by gradual evolution, some there are, who through a more fortunate and superior advantage or environment, have made the ascent faster and reached a more perfect and fixed state of morality, while there are others still, who have to be continually prodded to prevent a descending. Once let alone and retrogression sets in for there still lurks within them, a lingering taint of that ancestral brutality, ready to break out afresh when the opportunity presents.

Not until all the world shall have become educated to the full and deep meaning of the Fatherhood of God and the broth erhood of man, will the science and art of ethics become permanently and naturally fixed in our being and when that time shall come, if it ever does, we will surely be approaching a state of the highest and most perfect Christian civilization, possible of attainment.

